# An Organic Down-Converting Material for White-Light Emission from Hybrid LEDs

**DOI:** 10.1002/adma.201402661

**Published:** 2014-09-16

**Authors:** Neil J Findlay, Jochen Bruckbauer, Anto R Inigo, Benjamin Breig, Sasikumar Arumugam, David J Wallis, Robert W Martin, Peter J Skabara

**Affiliations:** WestCHEM, Department of Pure and Applied Chemistry, University of StrathclydeGlasgow, G1 1XL, UK; Department of Physics, SUPA, University of StrathclydeGlasgow, G4 0NG, UK; Plessey Semiconductor LtdTamerton Rd, Roborough, Plymouth, PL6 7BQ, UK

**Keywords:** hybrid light-emitting diodes (LEDs), energy down-converters, BODIPY, colorimetry

Materials suitable as active components in organic light-emitting diodes (OLEDs) have attracted widespread interest in recent years, as this technology develops towards replacing existing less efficient technologies, e.g., incandescent and fluorescent bulbs, in consumer applications such as solid-state lighting.^1^ To this end, both polymers^2^ and molecular structures^3^ have been exploited. While polymers offer high luminescence and solubility, they can suffer from problems associated with high polydispersity and batch-to-batch reproducibility. Molecular or oligomeric systems offer advantages such as monodispersity, synthetic reproducibility and, depending on structure and device fabrication requirements, they can be processed via vacuum deposition or solution processing techniques.^4^ Recently, Lee and co-workers have shown that higher device performance can be achieved via solution processing compared to vacuum-depositing the same emissive material.^5^

Although OLEDs offer significant advantages, such as low-cost manufacturing and flexibility, their use as white light sources is limited by their generally lower efficiency when compared with their inorganic LED counterparts. One interesting avenue to combat this issue is through a hybrid inorganic/organic LED architecture, where a blue emissive inorganic LED is coated with an organic material that has an absorbance band aligned with the emission wavelength of the inorganic structure.^6^ The organic material acts as an energy down-converter for the inorganic LED, converting some of the emitted high energy blue luminescence to lower energy yellow-red light that, when combined, delivers a high quality white output. The material system of choice for the inorganic LED is the III-nitride alloy system, which can emit light from the ultraviolet (UV), through the visible to infrared spectrum.^7^ Highly efficient blue emission is achieved by forming a quantum well structure of alternating thin layers of GaN and InGaN. Such hybrid inorganic/organic LED architectures offer the potential to combine the advantages of both technologies, for example, the excellent electronic properties of inorganic substrates and the broad, tunable emission of organic semiconductors.^8^ Furthermore, employing an organic material in place of traditional phosphors in hybrid LEDs would avoid an industry dependency on costly rare-earth containing materials as demand for solid-state lighting grows.^9^ The higher speed of response of the organic materials compared to the existing phosphors offers additional advantages for applications such as visible light communications.^10^

The 4,4-difluoro-4-borata-3*a*-azonia-4*a*-aza-*s*-indacene unit, hereafter referred to as BODIPY, has received widespread attention in recent years due to its attractive combination of properties including good solubility in a range of solvents, high absorptivity and high photoluminescence efficiency.^11^ As such, BODIPY is synonymous with numerous applications, such as biological labelling^12^ and sensors for ion detection.^13^ The use of BODIPY as the emissive component of a luminescent device is an interesting prospect due to its strong and tunable emissive properties; however, although the absorption band is intense, it is narrow and confined to around 500 nm.[11a] Hence, the combination of BODIPY with an absorbing partner unit, providing a more complex structure sensitive to higher energy wavelengths, is a useful method for developing designer organic molecules suitable for application in hybrid LED devices. Recently, we reported a novel family of linear oligofluorene-BODIPYs that are useful compounds for the down-conversion of UV light into visible light when drop-cast onto commercially available LEDs.^14^ In this communication, we present the synthesis of a novel organic energy down-converting material, **[BODFluTh]_2_FB**, and its application as the coemitter with a commercial blue LED. The resulting hybrid device demonstrates visible white light emission under a range of injection currents.

The molecule **[BODFluTh]_2_FB** was prepared via Stille coupling of compounds **1** and **2** in moderate yield and isolated as a bright orange powder (**Scheme** **1**). The structure of **[BODFluTh]_2_FB** was determined using standard analytical methods. The material was thermally stable, with 5% mass loss observed only upon heating to 395 °C. Additional thermal data was obtained following differential scanning calorimetry (DSC), with several thermal transitions evident, including values for *T*_g_ (128 °C), *T*_c_ (191 °C) and *T*_m_ (315 °C and 324 °C).

**Figure 3:**
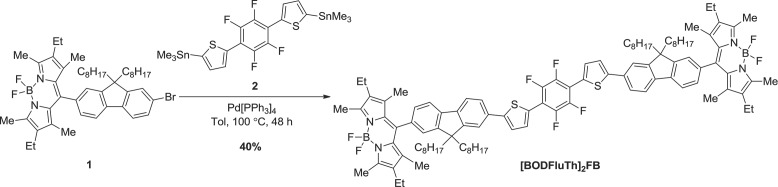
Scheme Synthesis of [BODFluTh]_2_FB.

In order to determine the suitability of **[BODFluTh]_2_FB** for the down-conversion of light, UV–vis absorption and emission spectra were recorded in dichloromethane solution (**Figure** **1**a). Two main absorption bands were evident: a broad band centered at 403 nm corresponding to a likely charge transfer process between the electron-deficient tetrafluorophenylene core and the adjacent thiophene-fluorene units, and a sharper peak at 525 nm, characteristic of the terminal BODIPY units.[11a] Initially, the fluorescence of **[BODFluTh]_2_FB** was determined by exciting the molecule at the absorption maximum corresponding to the broad charge transfer band of the core (403 nm), as this would identify whether energy transfer to the terminal BODIPY units was possible. An intense emission band at 550 nm was evident (not shown). Additionally, to take advantage of the broad nature of the absorption band at 403 nm, **[BODFluTh]_2_FB** was also excited at 440 nm – a typical wavelength of a blue inorganic LED – with the same emission band at 550 nm observed (Figure a), thus indicating that **[BODFluTh]_2_FB** would be suitable as a down-converting molecule for hybrid lighting devices. Furthermore, the electrochemical behavior of **[BODFluTh]_2_FB** was determined by cyclic voltammetry in dichloromethane solution (see Figure S1, Supporting Information and **Table 1**). Both oxidation and reduction processes were clearly evident, with a calculated HOMO-LUMO gap of 1.60 eV.

**Table 1:** Summary of the properties of [BODFluTh]_2_FB

λ_max_abs CH_2_Cl_2_^a^ [nm]	λ_max_em CH_2_Cl_2_^b^ [nm]	λ_max_abs encapsulated [nm]	λ_max_em encapsulated^c^ [nm]	PLQY CH_2_Cl_2_^d^, ^e^ [%]	PLQY encapsulated^d^ [%]
403, 525	550	404, 527	565	60	63

a) Recorded in CH_2_Cl_2_ at 10^−5^ M

b) Recorded in CH_2_Cl_2_ at 10^−6^ M with excitation at 440 nm

c) Excitation at 403 nm

d) Absolute values

e) Recorded as a 10^−5^ M solution.

**Figure 1:**
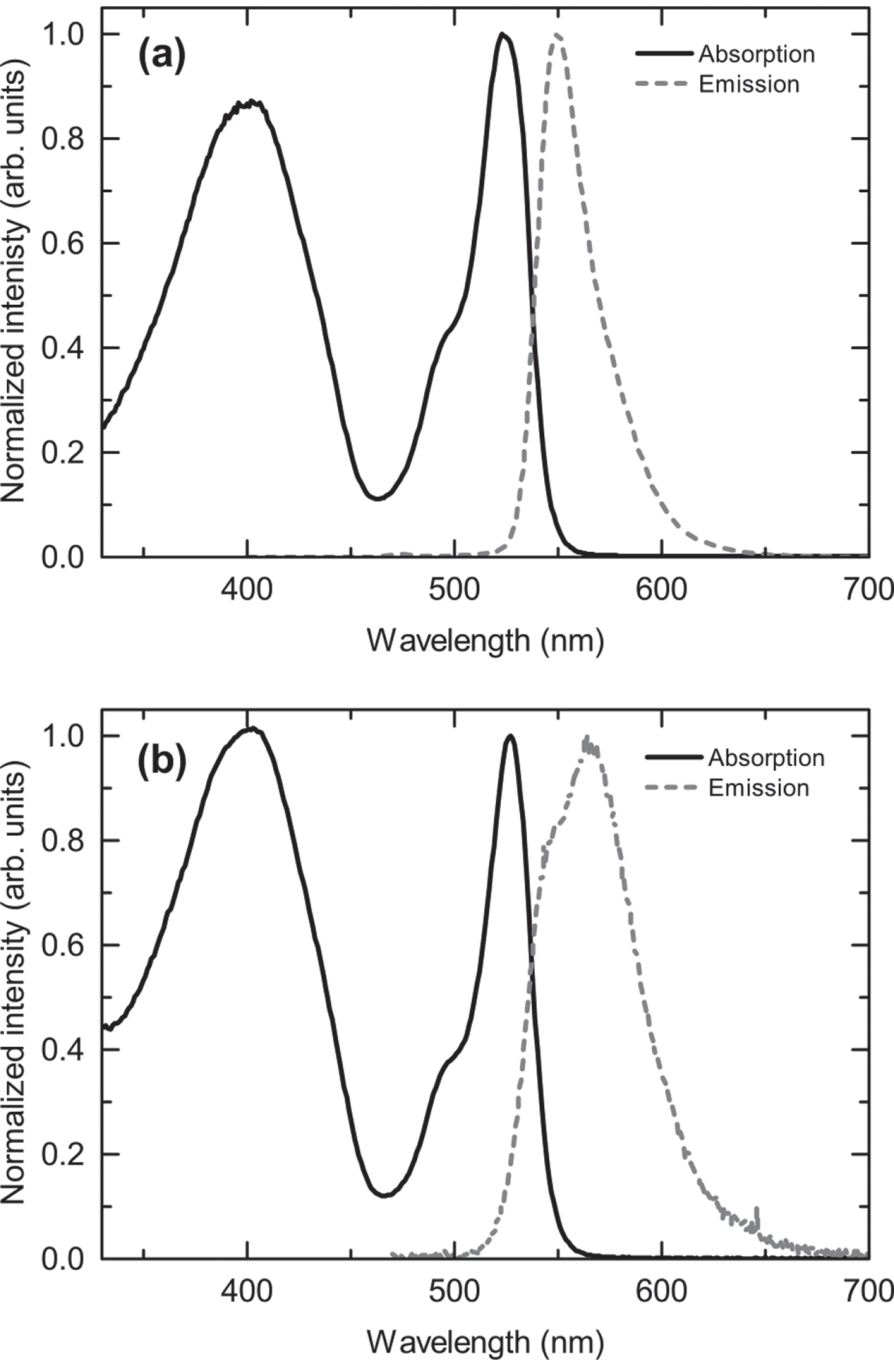
a) UV–vis absorption and emission spectra for [BODFluTh]_2_FB in dichloromethane solution (10^−5^ M). The solution was excited at 440 nm. b) UV–vis absorption and emission spectra for [BODFluTh]_2_FB encapsulated within the CHDV matrix. The encapsulated film was excited at 403 nm.

The deposition of organic emissive active layers within OLED devices typically involves either solution processing, for example spin-coating or drop-casting, or vacuum deposition.^15^ An alternative to these methods is encapsulation in a non-emissive matrix, a technique some of us demonstrated successfully in organic lasing applications.^16^ This provides several advantages, such as a low concentration of the bulk solution for deposition (i.e., 0.5–1% w/v), rapid curing of the encapsulant as opposed to extended annealing times and retention of the existing solution-state optical properties with minimal effects from annealing or other morphological changes. This last point also ensures a uniform and smooth down-conversion layer, free from micro-ordered sites that can affect the quality of light emitted and the overall efficiency of the device. A range of concentrations were applied with a 1% (w/v) solution of **[BODFluTh]_2_FB** in 1,4-cyclohexanedimethanol divinyl ether (CHDV), containing 0.5% of the photoacid generator (PAG) 4-octyloxy diphenyliodonium hexafluoroantimonate, proving optimal. The solution was deposited onto a standard glass microscope cover slip and exposure to UV light (254 nm) for 5 min effectively cured the matrix, encapsulating **[BODFluTh]_2_FB** as a green-colored film. We have applied CHDV as a matrix with other organic emissive materials to good effect, resulting in amorphous and robust films.^16^ It is worth noting that thin film work has been attempted on the pristine material **[BODFluTh]_2_FB**, but the films were of poor quality by drop-casting. Each attempt resulted in a poor, cracked film that wouldn't completely coat the LED, meaning that a significant amount of blue light leaked through. We therefore applied CHDV as a matrix because the neat material did not form good films, but a second reason was to suppress aggregation of **[BODFluTh]_2_FB** molecules and the potential for crystallization of the material with fluctuating temperature. No further encapsulation was necessary for our devices. Electronic absorption spectroscopy of the encapsulated film revealed two broad absorption peaks centered at 404 nm and 527 nm, values almost identical to the solution state measurements, as seen in Figure 1b. Furthermore, the emission spectra exhibited a desirable broadening of the band with a maximum at 565 nm and a high-energy shoulder at 547 nm. This is indicative of a small degree of aggregation, although the small shift of the emission maximum (just 15 nm) when compared to the solution state emission is a result of the dual effects of a low loading concentration and separation of the fluorophores by the inert matrix. Additional evidence for this comes from the photoluminescence quantum yields (PLQY) of the solution and encapsulated materials at 60% and 63%, respectively, indicating little difference between the optical properties of **[BODFluTh]_2_FB** whether in solution or encapsulated state.

To further study the color conversion capabilities **[BODFluTh]_2_FB** was integrated with the transparent CHDV layer and deposited on a fully-packaged blue LED. After curing, the organic material/matrix mixture formed a solid dome on top of the LED chip. The bare blue LED and the same LED encapsulated with the organic material (1% (w/v) **[BODFluTh]_2_FB** with 1% PAG) are shown in **Figure** **2**a and c, respectively. As can be seen in Figure 2c the entire cup, containing the LED with its wire bond, is fully encapsulated with the organic material. Photographs of the same LED under a drive current of 25 mA are shown in Figure 2b and 2d. Compared with the bare blue LED, the encapsulated LED with the organic converter shows the desired yellow emission in the vicinity of the LED chip. By applying a range of concentrations of **[BODFluTh]_2_FB** (0.5%, 1% and 4%), we found that the 1% formulation gave the best results in terms of tuning the chromaticity to white light (see Figure S2, Supporting Information). A photograph of the white LED from a distance, matching what would be perceived by the eye, is shown in Figure 2g. To fully appreciate the concept of down-conversion in this work, Figure S3 in the Supporting Information shows the alignment of energy levels required for the LED chip and organic to work together and achieve white light emission.

**Figure 2:**
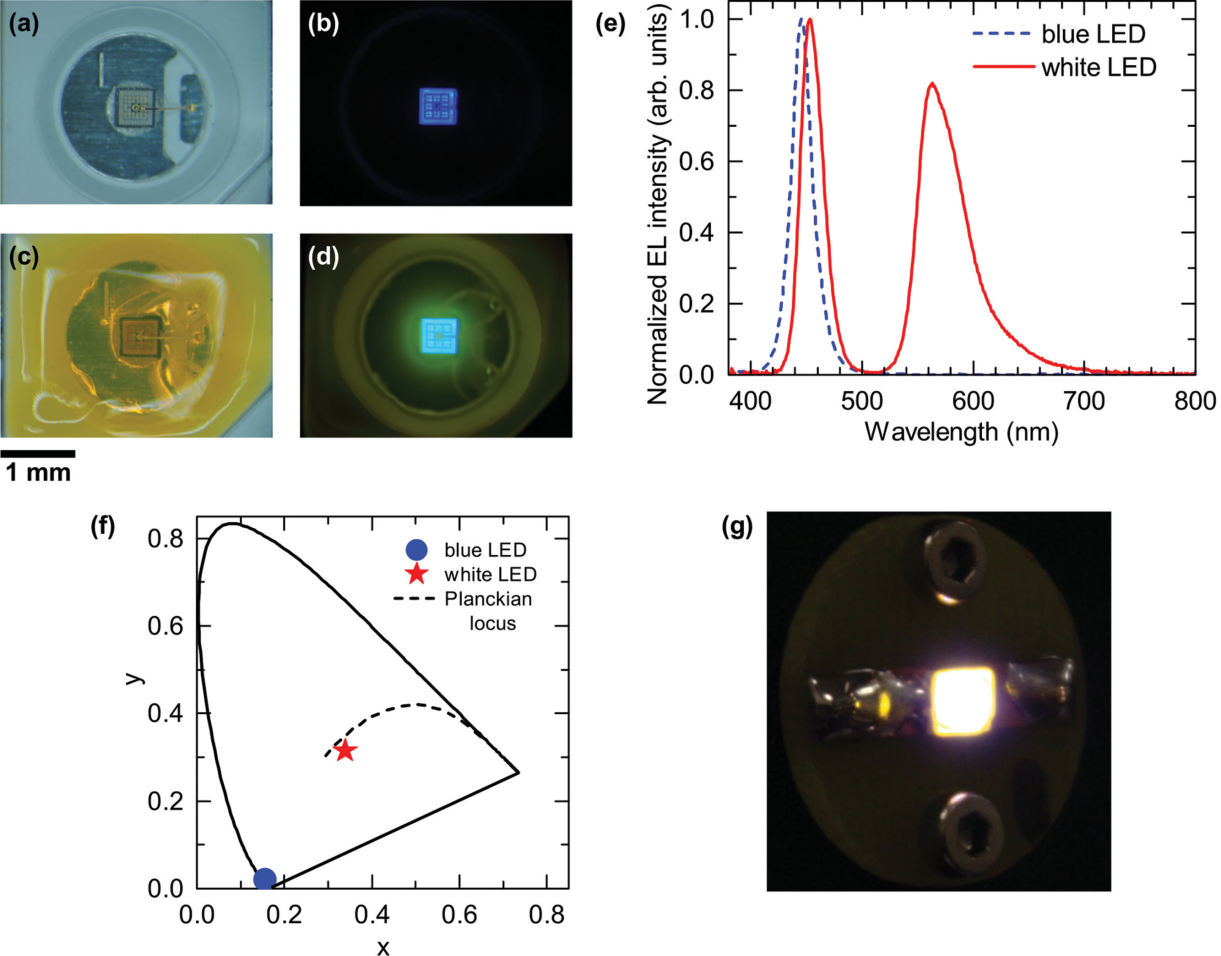
a–d) Photographs of the (a,b) bare blue LED and (c,d) the same LED encapsulated with the organic material (1% (w/v) [BODFluTh]_2_FB with 1% PAG) with the LED (a,c) switched off and (b,d) under a forward current of 25 mA. e) EL spectra of the blue LED before and after encapsulation with the 1% organic wavelength converter. The spectra are normalized to the blue LED peak intensity. f) Chromaticity diagram (CIE 1931) showing the coordinates of the same LED. Additionally, the Planckian locus (dashed line) is shown as a reference. g) A photograph of the white LED from a distance.

For the electroluminescence (EL) measurements the LEDs were placed inside an integrating sphere and a forward current of 25 mA was applied. Figure e displays the EL spectra of the LED before and after deposition of the 1% organic wavelength converter. Both spectra are normalized to the blue emission peak. The spectrum of the bare blue LED consists of a single emission peak at about 445 nm corresponding to electron-hole recombination in the quantum well structure of the LED.^17^ After deposition of the 1% organic wavelength converter, a second emission peak centered around 560 nm appears, resulting from partial absorption of the blue light by the organic material followed by yellow emission. The asymmetry of the yellow emission is due to self-absorption by the BODIPY dye, which is the yellow emitter, causing the steeper drop on the lower wavelength side.[^11a^] Also, the blue emission peak shifts slightly to longer wavelength due to selective absorption by the **[BODFluTh]_2_FB** molecule.

The calibrated integrating sphere collects all of the emitted light making an absolute intensity measurement possible. The luminous efficacy (lm/W) describes the efficiency of the power conversion and is defined as the ratio of the luminous flux and electrical input power. Most importantly the luminous flux accounts for the sensitivity of the human eye. The efficacy of the bare blue LED is 3.2 lm/W, and increases by a factor greater than four to 13.6 lm/W after adding the 1% organic converter. This increase is due to the additional contribution by the emission peak in the yellow region of the spectrum. The EL spectra were corrected for the system response and then used to determine the chromaticity coordinates (*x*, *y*) in the Commission Internationale de l’Eclairage (CIE) 1931 color space chromaticity diagram and the correlated color temperature (CCT). Figure 2f displays the chromaticity coordinates of the bare blue LED, (0.16, 0.02), and the same LED coated with the 1% organic converter, which are (0.34, 0.31). The coordinates of the blue LED are located very close to the perimeter of the chromaticity diagram where monochromatic light sources are found. After adding the 1% solution of **[BODFluTh]_2_FB** to the same LED the coordinates move towards the center of the diagram, where white light is located, and also closer to the Planckian locus, which corresponds to light emitted from a black body radiator at various color temperatures. The color temperature is a measure of the “whiteness”, i.e., warm or cold white, of a white light source. The CCT of the LED encapsulated with the 1% organic converter is calculated to be 5137 K, which makes the perception of the light as cool white. Although the LED appears white, its color rendering properties are not ideal. This can be explained by the shape of the spectrum of the LED (Figure 2e), which consists of two distinct peaks, one in the blue and one in the yellow region. These peaks, however, are separated by a considerable gap in the green region. The human eye is most sensitive in the green spectral region, and this part of the spectrum is missing. The gap below ca. 550 nm is due to self-absorption of the BODIPY and an inherent property affecting the performance of **[BODFluTh]_2_FB** as a color converter.

To investigate the uniformity of the light emission, EL hyperspectral imaging was performed on the white LED coated with the 1% organic converter, which spatially and spectrally records the emitted light allowing us for example to generate two-dimensional maps of the intensity of different EL emission peaks.^18^ The results are displayed in Figure S4 in the Supporting Information. The EL maps clearly demonstrate that the blue emission from the LED is localized to the area of the LED die. The yellow emission from the organic material, however, can be observed from the entire encapsulant and the emission is isotropic in nature.

For an estimate of the device lifetime, an encapsulated LED (1% (w/v) **[BODFluTh]_2_FB** with 1% PAG) was switched on at 25 mA once a day for the duration of the measurement (5 s) over a period of 28 days. During the course of the experiment, no significant change of the yellow emission band, chromaticity coordinates or CCT was observed (see Figure S5, Supporting Information). A more strenuous examination was conducted by switching the device on continuously for a period of several hours (see Figure S6, Supporting Information). Under constant current (25 mA), the intensity of the yellow band dropped off, while the intensity of the blue emission peak remained constant (Figure S6) making the LED appear more blue over time. Additionally, lowering the drive current (5 mA) did not serve to enhance the lifetime measurement (Figure S7, Supporting Information). While it is possible that heat generated from the blue LED could contribute to a detrimental effect on **[BODFluTh]_2_FB** (the decomposition temperature was recorded as 395 °C in Ar, while the material has a *T*_g_ at 128 °C), it is also possible that any excess heat could affect the encapsulating matrix. As such, separating the blue LED and the encapsulated organic layer should serve to reduce this effect and enhance the lifetime of the functioning device. This has been investigated by depositing the encapsulated organic material on a glass slide placed 5 millimeters above the LED. As seen in Figure S8 in the Supporting Information, the efficacy decreases by less than 10% after about 200 h of continuous operation at 25 mA and the CCT and chromaticity coordinates remained almost constant.

In summary, a novel, energy down-converting light emitter has been investigated as an effective color converter for application in hybrid inorganic/organic white LEDs. Using CHDV as an encapsulating, UV-curable matrix, a dilute solution (1% w/v) of organic converter material was deposited on a blue LED and produced an additional, strong emission in the yellow spectrum (at around 560 nm) resulting from partial absorption by the organic material and re-emission at a higher wavelength. Further analysis showed that the chromaticity coordinates are very close to the center of the chromaticity diagram where white light is located and that the color temperature is that of cold/cool white light. A fourfold increase in luminous efficacy was also observed compared with the bare blue LED, indicating the promise of this technique to realize white light emitting hybrid devices. Further efforts are ongoing to optimize the compatibility of LED and organic down-converting layer to enhance the quality of light emitted.

## Experimental Section

*Synthesis of **[BODFluTh]_2_FB***: Compound **1** (199 mg, 0.258 mmol, 2.2 eq.) and compound **2** (75 mg, 0.117 mmol, 1.0 eq.) were charged to a reaction flask and Pd[PPh_3_]_4_ (14 mg, 0.012 mmol, 0.1 eq.) was added. The contents of the reaction flask were evacuated and purged with Ar. Anhydrous toluene (15 mL) was then added and the reaction mixture heated to 100 °C for 48 h. After this time, the mixture was cooled to r.t. and diluted with toluene (30 mL), before being washed with water (50 mL). The aqueous layer was extracted with CH_2_Cl_2_ (2 × 40 mL) and all organic layers combined. The combined organic layers were washed with water (2 × 50 mL) and brine (2 × 50 mL), then dried over MgSO_4_ and concentrated under vacuum. The resultant residue was purified by silica-gel column chromatography, eluting with 40–60% CH_2_Cl_2_/hexane, afforded **[BODFluTh]_2_FB** as a bright orange powder (80 mg, 40%); TGA: 5% mass loss at 395 °C; *T*_g_ = 128 °C, *T*_c_ = 191 °C, *T*_m_ = 315 and 324 °C; ^1^H NMR (400 MHz, CD_2_Cl_2_, *δ*): 7.88 (d, *J* = 8.0 Hz, 2H, ArH), 7.84 (d, *J* = 8.0 Hz, 2H, ArH), 7.76–7.74 (m, 4H, ArH), 7.70 (d, *J* = 1.6 Hz, 2H, ArH), 7.54 (d, *J* = 3.6 Hz, 2H, ArH), 7.35 (s, 2H, ArH), 7.30 (dd, *J* = 7.6 and 1.6 Hz, 2H, ArH), 2.51 (s, 12H, CH_3_), 2.34 (q, *J* = 7.6 Hz, 8H, CH_2_), 2.15–2.01 (m, 8H, CH_2_), 1.39 (s, 12H, CH_3_), 1.21–0.98 (m, 52H, CH_2_, CH_3_), 0.81 (t, *J* = 7.6 Hz, 12H, CH_3_), 0.74–0.61 (m, 8H, CH_2_); ^13^C NMR (100 MHz, CDCl_3_, *δ*): 153.1, 151.5, 151.4, 140.9, 140.6, 140.3, 138.1, 134.1, 132.41, 132.35, 131.1, 130.3, 126.8, 124.5, 123.0, 122.6, 120.1, 119.8, 55.3, 40.0, 31.3, 29.5, 28.9, 28.7, 23.4, 22.1, 16.5, 13.8, 13.3, 11.7, 11.5; MALDI (*m/z* (%)) 1695 (100); Anal calcd for C_106_H_128_B_2_F_8_N_4_S_2_: C 75.07, H 7.61, N 3.30, S 3.78; found: C 74.97, H 7.58, N, 3.80, S, 3.85.
