# Supplementary material for: An Organic Down-Converting Material for White-Light Emission from Hybrid LEDs
Source: Adv Mater. 2014 Sep 16;26(43):7290–4. doi: 10.1002/adma.201402661 (PMC4241046; doi:10.1002/adma.201402661)
Supplement: Supplementary file 1 — Supplementary [file adma0026-7290-SD1.pdf]

Copyright WILEY-VCH Verlag GmbH & Co. KGaA, 69469 Weinheim, Germany, 2014.

ADVANCED MATERIALS

Supporting Information

for *Adv. Mater.*, DOI: 10.1002/adma.201402661

An Organic Down-Converting Material for White-Light Emission from Hybrid LEDs

Neil J. Findlay, Jochen Bruckbauer, Anto R. Inigo, Benjamin Breig, Sasikumar Arumugam, David J. Wallis, Robert W. Martin, and Peter J. Skabara**

Supporting Information

An Organic Down-Converting Material for White-Light Emission from Hybrid LEDs

By Neil J. Findlay,[a] Jochen Bruckbauer,[b] Anto R. Inigo, [a] Benjamin Breig, [a] Sasikumar Arumugam, [a] David J. Wallis,[c] Robert W. Martin [b] and Peter J. Skabara [a]**

[a] WestCHEM, Department of Pure and Applied Chemistry, University of Strathclyde, Glasgow, G1 1XL, UK

[b] Department of Physics, SUPA, University of Strathclyde, Glasgow, G4 0NG, UK

[c] Plessey Semiconductor Ltd, Tamerton Rd, Roborough, Plymouth, PL6 7BQ, UK

Table of contents:

General experimental………………………………………………………………….. 2

Synthesis of compound **2**……………………………………………………………… 4

Figure S1: Cyclic voltammetry of compound **[BODFluTh]$_2$FB**……………………… 5

Figure S2: Electroluminescence and chromaticity diagram for encapsulated LEDs at a range of concentrations……………………………………………………………… 6

Figure S3: Matching of the emission and absorption characteristics of the hybrid device 7

Figure S4: Electroluminescence hyperspectral imaging of the LED coated with 1% of the organic converter……………………………………………………………… 8

Figure S5: Lifetime measurement for encapsulated LED – 25 mA, once per day……….. 10

Figure S6: Lifetime measurement for encapsulated LED – 25 mA, continuous………… 11

Figure S7: Lifetime measurement for encapsulated LED – 5 mA, continuous…………... 12

Figure S8: Lifetime measurement for encapsulated LED utilizing “air –gap” – 25 mA, continuous……………………………………………………………………… 13

General Experimental

Compound **1**[1] and 1,4-bis(2-thienyl)-2,3,5,6-tetrafluorobenzene[2] were prepared according to literature methods. Tetrakis(triphenylphosphine)palladium(0) ($Pd(PPh_3)_4$) was prepared prior to use and stored under nitrogen. Unless otherwise stated, all other reagents were sourced commercially and used without further purification. Dry solvents were obtained from a solvent purification system (SPS 400 from Innovative Technologies) using alumina as the drying agent. ^{1}H and ^{13}C NMR spectra were recorded on either a Bruker DRX 500 apparatus at 500.13 and 125.76 MHz, or a Bruker Avance DPX400 apparatus at 400.13 and 100.6 MHz. Chemical shifts are given in ppm; all J values are in Hz. Elemental analyses were obtained on a Perkin-Elmer 2400 analyser. MS LDI-TOF spectra were run on a Shimadzu Axima-CFR spectrometer (mass range 1-150000 Da). Thermogravimetric analysis (TGA) was performed using a Perkin-Elmer Thermogravimetric Analyzer TGA7 under a constant flow of helium. Melting points were taken using a TA instruments DSC QC1000 Differential Scanning Calorimeter, and are uncorrected.

Cyclic voltammetry (CV) measurements were performed on a CH Instruments 660A electrochemical workstation with iR compensation using anhydrous dichloromethane as the solvent. The electrodes were glassy carbon, platinum wire and silver wire as the working, counter and reference electrodes, respectively. All solutions were degassed (Ar) and contained monomer substrates in concentrations of ca. 10^{-4} M, together with n-Bu4NPF$_6$ (0.1 M) as the supporting electrolyte. All measurements are referenced against the $E_{1/2}$ of the Fc/Fc^+ redox couple. Absorption spectra were recorded on a Shimadzu UV 2700 instrument. Photoluminescence measurements were recorded using a Perkin-Elmer LS 50 B fluorescence spectrometer in a quartz cuvette (path length 10 mm). Absolute photoluminescence quantum yield measurements were measured according to the de Mello method by using a calibrated integrating sphere attached to an USB 2000 spectrometer and Gooch & Housego

spectrometer. Excitation light was chosen from a Quartz Tungsten Halogen lamp by using a Gooch & Housego spectrometer and the emission light was collected by Ocean optics USB 2000 spectrometer. Measurements were performed in air.

The light-emitting diodes (LEDs) used for the color conversion process were supplied by Plessey Semiconductors Ltd, Roborough, UK. They consist of a fully packaged blue-emitting GaN-on-Si LED with an emission wavelength of about 445 nm. The forward current of the LEDs is 25 mA. Electroluminescence (EL) measurements of the LEDs were carried out at room temperature using a Keithley 236 source measure unit as a current source. For the light collection the LEDs were placed inside a 25 cm diameter integrating sphere (Labsphere illumina®plus 600/610) coupled to a spectrometer (CDS-610, wavelength accuracy <0.5 nm) using a 600 μm diameter single core fiber cable. The entire system is corrected for its response using a calibrated spectral and luminous flux standard (SCL-600). Chromaticity coordinates (CIE 1931) and correlated color temperature (CCT) are calculated from the response-corrected spectra.

*Synthesis of compound **2***

1,4-Bis(2-thienyl)-2,3,5,6-tetrafluorobenzene (300 mg, 0.954 mmol, 1.0 equiv.) was dissolved in anhydrous THF (30 mL) and cooled to -80 °C where *n*-butyllithium (2.34 M, 0.82 mL, 1.922 mmol, 2.01 equiv.) was added slowly to maintain low temperature. The reaction mixture was stirred at low temperature for 1.5 h, then trimethyltin chloride (1.0M in THF, 2.87 mL, 2.868 mmol, 3.0 equiv.) was added. The reaction mixture was held at low temperature for 2 h, then allowed to warm to room temperature overnight. After this time, the mixture was diluted with dichloromethane (80 mL) and washed with water (100 mL). The aqueous layer was further extracted with dichloromethane (2 × 70 mL) and all organic layers combined. These combined organic layers were washed further with water (2 × 100 mL) and brine (100 mL), then dried over $MgSO_4$ and concentrated under vacuum. The resultant yellow powder was dried under vacuum overnight and subsequently used in the synthesis of **[BODFluTh]$_2$FB** without further purification.

Figure S1: Cyclic voltammogram of **[BODFluTh]$_2$FB** in CH_2Cl_2.

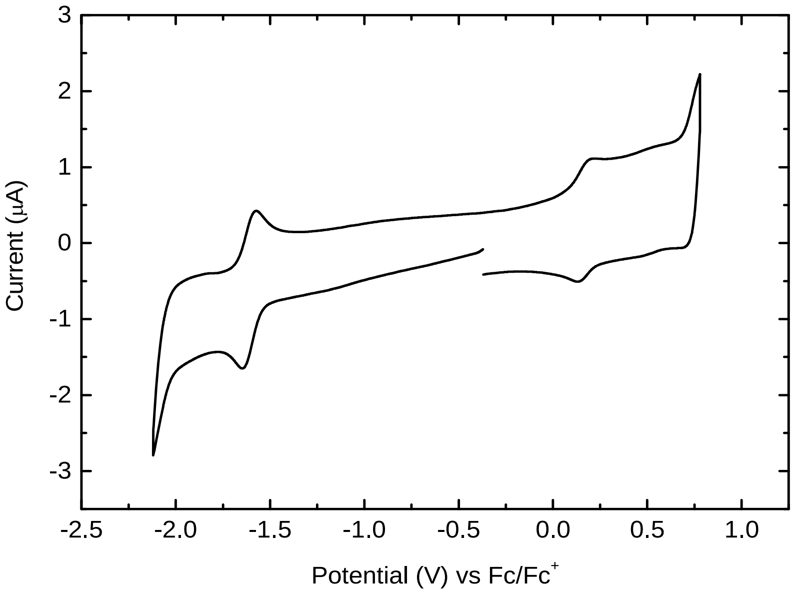

Glassy carbon working electrode, Ag wire reference electrode and Pt counter electrode, in CH_2Cl_2 at a scan rate of 100 mVs^{-1}, 0.1 M $TBAPF_6$ as supporting electrolyte. The data is referenced to the Fc/Fc+ redox couple.

Figure S2. (a) EL spectra of the blue LED before and after encapsulation with the 0.5%, 1% and 4% organic wavelength converter. **(b)** Chromaticity diagram (CIE 1931) showing the coordinates of the same LEDs.

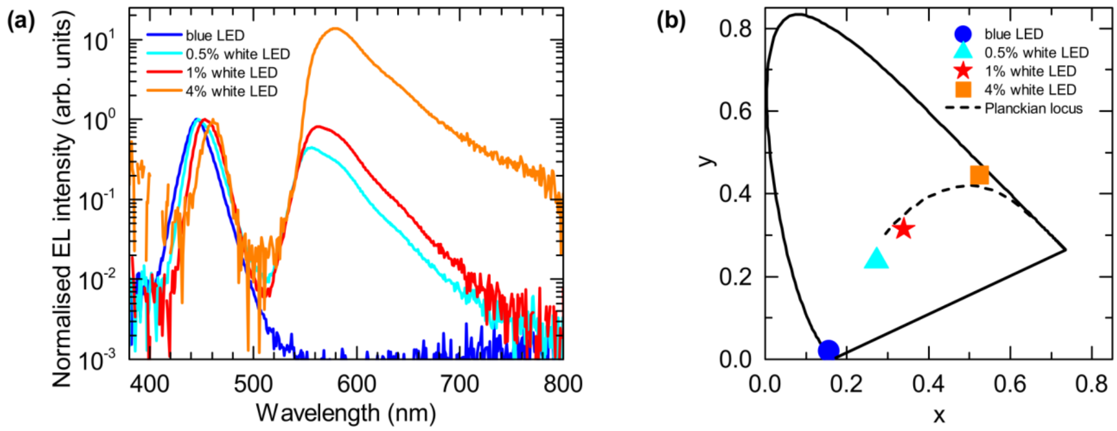

To study the color conversion capabilities of **[BODFluTh]$_2$FB** different concentrations of the organic converter were integrated into a transparent matrix (CHDV). The studied concentrations were 0.5%, 1% and 4%. Electroluminescence (EL) spectra, recorded using an integrating sphere, of these LEDs coated with these concentrations are shown in **Figure S2**. The spectra are normalized to the peak intensity of the blue LED peak. Additionally, the Planckian locus (dashed line) is shown as a reference.

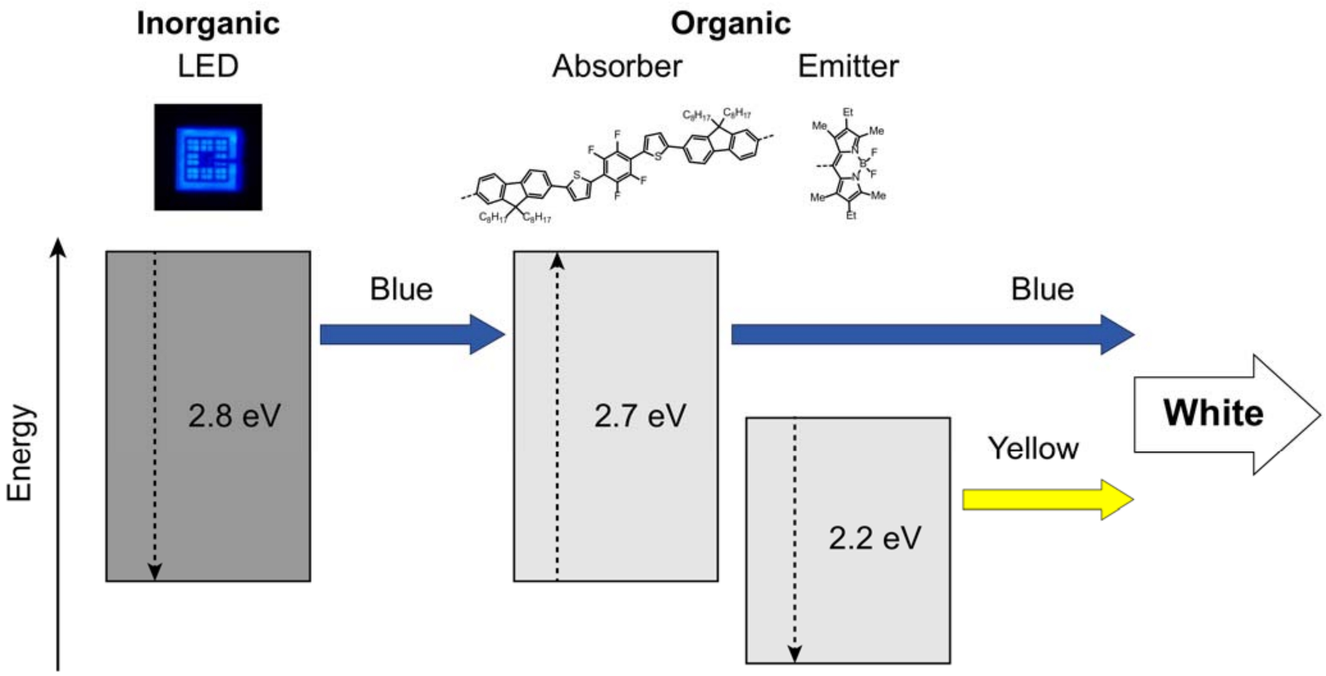

Figure S3. Schematic representing the energies of absorbed and emitted light (blue emission from the LED chip, absorption and then yellow emission from the organic), providing resultant white light.

Figure S4. (a) Photograph of the LED with 1% of the organic material deposited on the top. EL hyperspectral data set (LED operated under a constant forward current of 25 mA): **(b)** real color image; **(c)** integrated EL intensity map of the blue LED emission peak (400-500 nm) and **(d)** integrated EL intensity map of the yellow emission peak (500-750 nm) of the organic material. **(e)** Typical spectrum of the blue and yellow emission from two pixels as marked in (c) and (d).

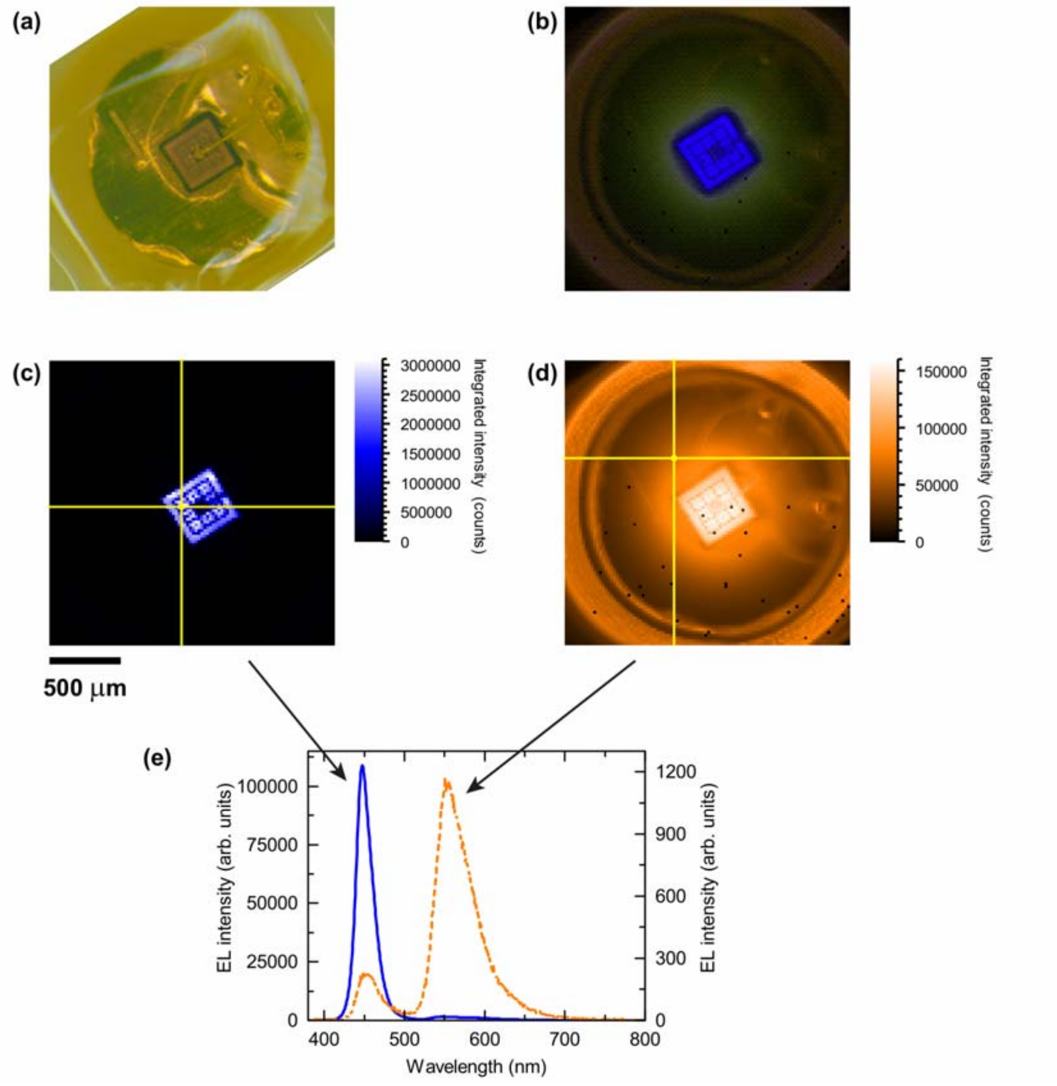

Figure S4 displays the result of EL hyperspectral imaging of the LED coated with 1% of the organic converter under a constant forward current of 25 mA. EL mapping was performed by collection the light through a pinhole while scanning the sample below the point of focus.

The luminescence was spatially (resolution 3μm) and spectrally (< 1 nm) resolved. **Figure S4(a)** shows a photograph of the LED from which the EL map was collected. A real color image calculated from the hyperspectral data set was generated and is displayed in **Figure S4(b)**. Integrated EL intensity images of the blue LED emission peak (400-500 nm) and the yellow emission peak (500-750 nm) from the organic converter are shown in **Figures S4(c)** and **(d)**. It can clearly be observed that the emission from the LED is localized to the LED die, whereas emission from the organic material is observed everywhere due to isotropic nature of the emission. A typical spectrum of the blue and yellow emission is shown in **Figure S4(e)**.

Figure S5. (a) EL spectra; **(b)** CIE chromaticity coordinates of white LED driven at 25 mA for the duration of the measurement once a day for 28 days.

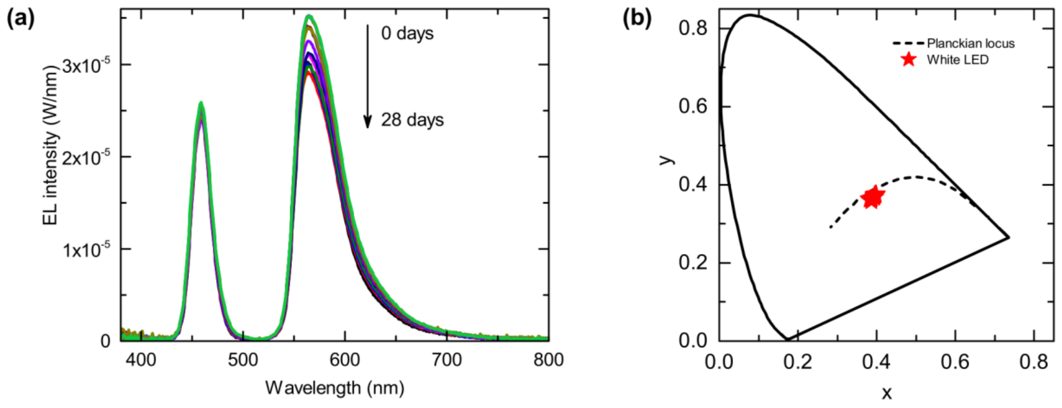

To investigate the lifetime of the devices an encapsulated LED (1% (w/v) **[BODFluTh]$_2$FB** with 1% PAG) was switched on at a current of 25 mA once a day for the duration of the measurements (several seconds) over a period of 28 days. The EL spectra and chromaticity coordinates are shown in **Figure S5(a)** and **(b)**, respectively. Besides a small change in the intensity of the yellow emission peak no significant change was observed. The chromaticity coordinates and the CCT remained almost constant.

Figure S6. (a) EL spectra; **(b)** CIE chromaticity coordinates and **(c)** luminous efficacy of white LED driven at 25 mA for several hours.

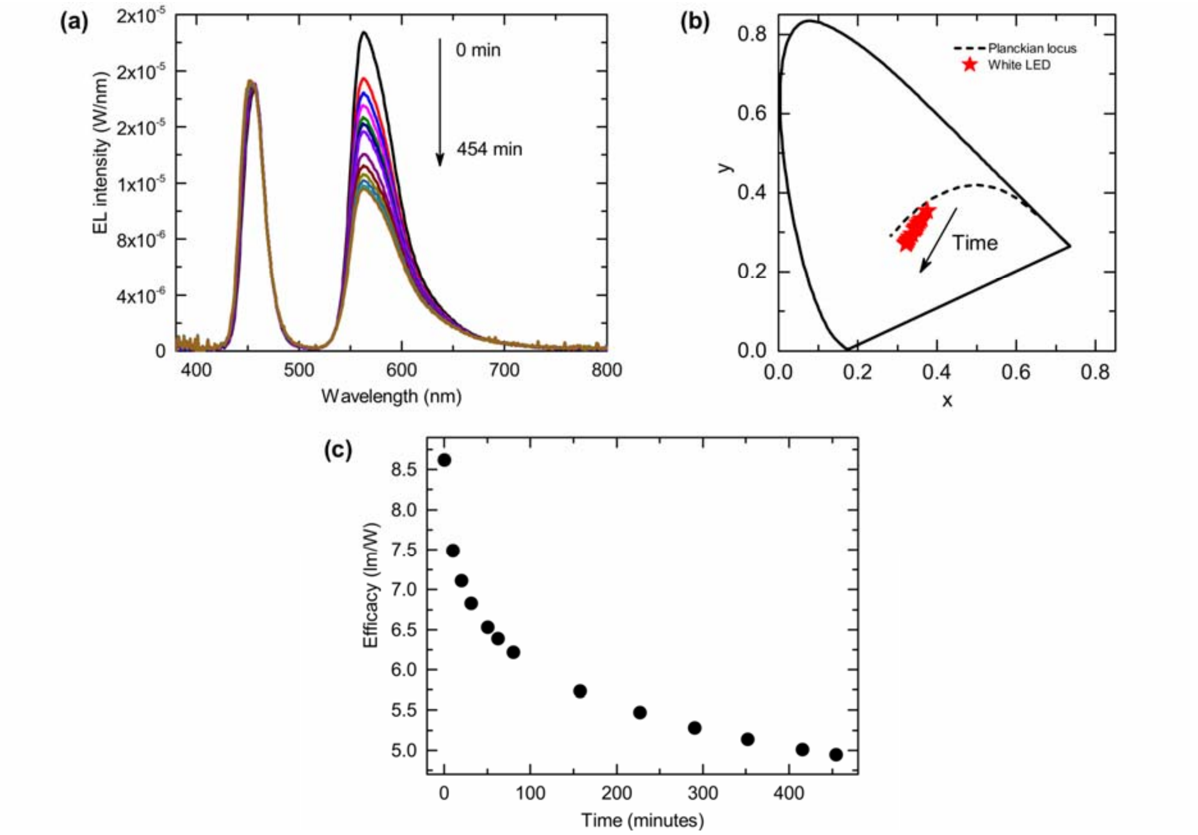

For a more long term examination the LED was continuously switched on at 25 mA for several hours and measurements were taken at certain intervals. The results are shown in **Figure S6** where EL spectra, chromaticity coordinates and the luminous efficacy are displayed. Over time the intensity of the yellow emission band dropped off, while the blue emission band stayed constant. This leads to the light appearing more blue over time, as seen in the chromaticity coordinates, which moved towards the perimeter of the diagram where blue light is located. Furthermore, the luminous efficacy also exponentially decreases with time as the contribution of the yellow emission peak is reduced.

Figure S7. **(a)** EL spectra; **(b)** CIE chromaticity coordinates and **(c)** luminous efficacy of white LED driven at 5 mA for several hours.

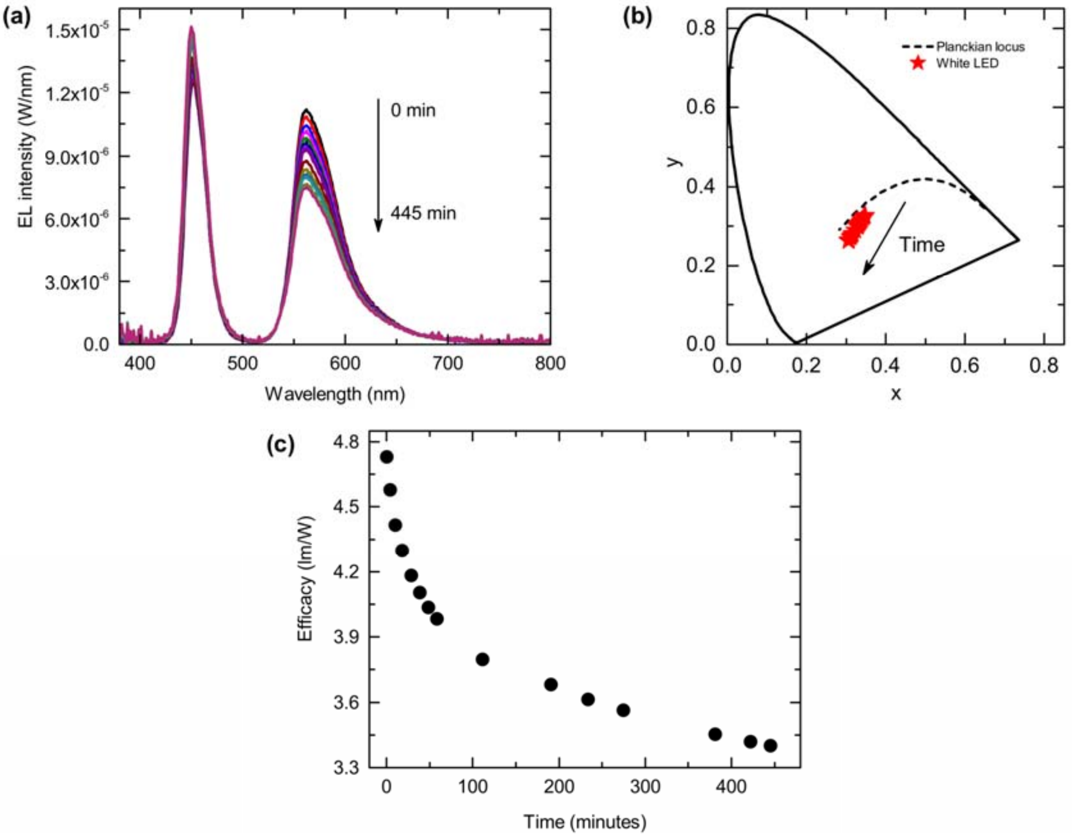

The same measurement was performed at a lower current of 5 mA. The results are shown in **Figure S7**. As expected the intensity of the yellow peak decreases much slower with time compared with the measurement at 25 mA. Also the luminous efficacy and chromaticity coordinates are affected less strongly.

Figure S8. (a) EL spectra; **(b)** CIE chromaticity coordinates and **(c)** luminous efficacy of white LED driven at 25 mA for several days where the organic material was deposited on a glass slide placed 5 millimeters above the blue LED.

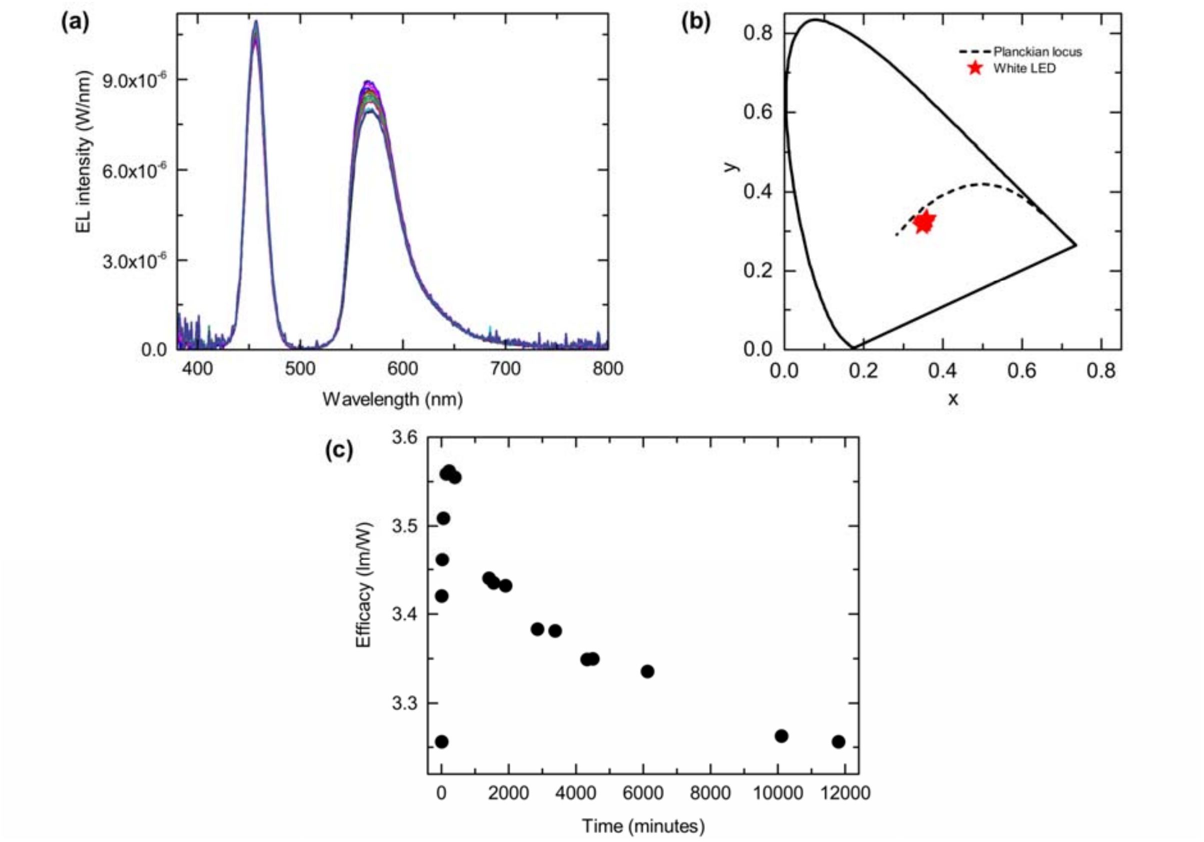

For investigating the influence of the heat generated by the blue LED on the encapsulated organic material **[BODFluTh]$_2$FB** was deposited on a glass slide and placed 5 millimeters above the LED, effectively separating the LED from the organic material. The LED was continuously operated at a current of 25 mA for several days. The results, namely the EL spectra, chromaticity coordinates and luminous efficacy are shown in **Figure S8** After a slight increase in intensity of the yellow emission peak in the first two hours the intensity of this peak marginally decreased over the next several days. This has an effect on the efficacy as shown in **Figure S8 (c)**, which decreased after an initial increase. However, even after almost 200 h of continuous operation the efficacy decreases by less than 10%. The chromaticity coordinates remained almost constant as seen in **Figure S8 (b)**. The CCT also

almost remained the same. Compared with the results shown in **Figures S6** and **S7** this illustrates that the heat generated by the LED has a detrimental effect on the organic material when in close contact.

References

1. N. J. Findlay, C. Orofino-Peña, J. Bruckbauer, S. E. T. Elmasly, S. Arumugam, A. R. Inigo, A. L. Kanibolotsky, R. W. Martin, P. J. Skabara, *J. Mater. Chem. C* **2013**, *1*, 2249.
2. D. J. Crouch, P. J. Skabara, J. E. Lohr, J. J. W. McDouall, M. Heeney, I. McCulloch, D. Sparrowe, M. Shkunov, S. J. Coles, P. N. Horton, M. B. Hursthouse, *Chem. Mater.* **2005**, *17*, 6567.
